# Polydopamine-mediated curcumin-functionalized electroactive nanofibrous scaffold accelerates infected wound healing via multifactorial microenvironment remodeling

**DOI:** 10.3389/fbioe.2026.1959216

**Published:** 2026-10-14

**Authors:** Mintao Tian, Yuntian Bai, Zhongdi Liu, Ming Cheng, Mingyue Song, Jiahua Zhou, Wei Guo, Ming Li, Jianbo Xue

**Affiliations:** 1 Department of Plastic Reconstructive Surgery and Hand Microsurgery, Ningbo No. 6 Hospital, Ningbo, Zhejiang, China; 2 Ningbo Clinical Research Center for Orthopedics, Sports Medicine and Rehabilitation, Ningbo, Zhejiang, China; 3 Trauma Medicine Center, Peking University People’s Hospital, Beijing, China; 4 Key Laboratory of Trauma and Neural Regeneration, Peking University, Ministry of Education, Beijing, China; 5 National Center for Trauma Medicine, Beijing, China; 6 Emergency Department, Peking University People’s Hospital, Beijing, China

**Keywords:** bacterial SOS response, curcumin, electrospinning, infected wound, sequential drug release

## Abstract

Effective remodeling of the wound microenvironment is critical for achieving rapid and high-quality skin regeneration. However, current therapeutic strategies still struggle to simultaneously address the multiple pathological features present in complex wounds, including persistent inflammation, oxidative stress, bacterial infection, and impaired angiogenesis. In this study, we constructed a multifunctional electroactive nanofibrous scaffold, PCL/CNT-PDA@Cur, by functionalizing the surface of a polycaprolactone/carbon nanotube electrospun matrix with polydopamine (PDA)-mediated curcumin (Cur) loading. This surface functionalization is attributed to the π–π stacking and hydrogen bonding interactions between the catechol groups of PDA and the curcumin molecules. The resulting scaffold mimics the structure of the native extracellular matrix (ECM), exhibits enhanced hydrophilicity, stable electrical conductivity, and sustained curcumin release properties. *In vitro* experiments demonstrated that the PCL/CNT-PDA@Cur scaffold significantly promoted the proliferation and migration of L929 fibroblasts, upregulated the expression of collagen types I and III, and protected cells against oxidative stress-induced apoptosis. The scaffold also enhanced the tube-forming capacity of HUVECs and the expression of angiogenesis-related genes, while effectively scavenging intracellular reactive oxygen species. Moreover, the scaffold was able to drive macrophage polarization toward the anti-inflammatory M2 phenotype. In addition, it exhibited pronounced antibacterial activity against *Staphylococcus aureus* and *Escherichia coli* by disrupting bacterial membrane integrity. In the rat full-thickness skin defect infection model, the PCL/CNT-PDA@Cur scaffold effectively controlled wound infection, significantly accelerated wound closure, promoted granulation tissue formation, collagen deposition, and neovascularization, and facilitated the regeneration of skin appendages such as hair follicles. In summary, the PDA-mediated curcumin-loaded electroactive scaffold constructed in this study integrates multiple functions—including antioxidant, antibacterial, immunomodulatory, and pro-angiogenic activities—into a single platform, and preliminarily reveals a potential mechanism involving interference with the bacterial SOS response network through downregulation of RecA and LexA along with upregulation of SulA gene expression, thereby providing a new strategy and experimental basis for the treatment of complex infected wounds.

## Graphical Abstract

**Figure:**
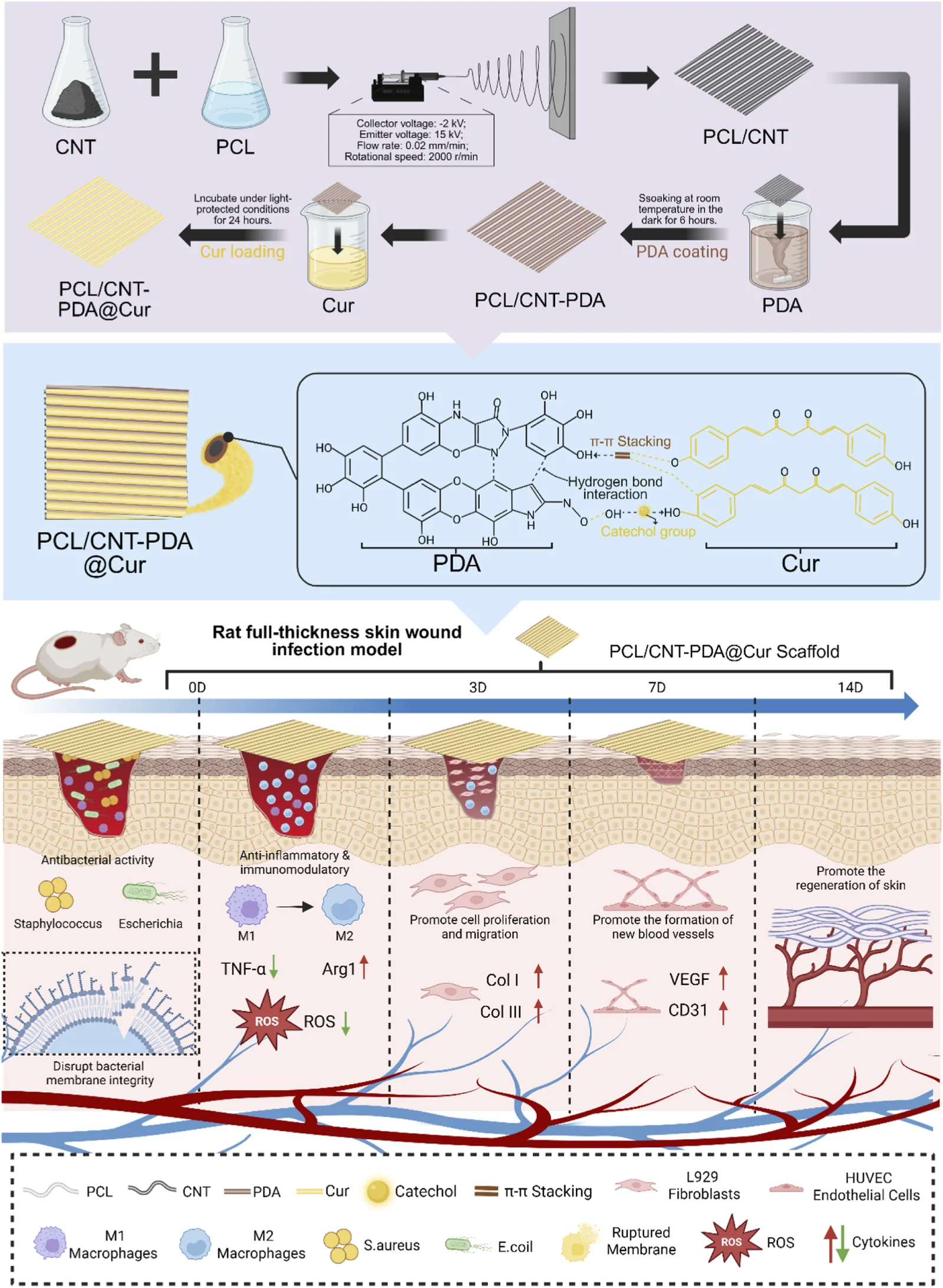
Polydopamine-mediated curcumin-functionalized electroactive nanofibrous scaffolds for accelerated infected wound healing via multifactorial microenvironment remodeling.

## 1 Introduction

As the largest organ of the human body, the skin performs multiple vital physiological functions, including protection, sensation, thermoregulation, and metabolism (Nguyen and Soulika, 2019). Wound healing following skin injury is a complex and precisely regulated biological process involving four overlapping phases: hemostasis, inflammation, proliferation, and remodeling (Mamun et al., 2024). However, full-thickness skin defects caused by trauma, burns, or chronic diseases—particularly large or non-healing wounds—often experience impaired healing due to disturbances in the wound microenvironment. These disturbances manifest as persistent inflammation, excessive accumulation of reactive oxygen species (ROS), bacterial infection, and impaired angiogenesis, leading to prolonged healing or even non-closure, thereby imposing a heavy burden on patients and healthcare systems (Milner et al., 2021). Therefore, the development of multifunctional dressings capable of effectively modulating the wound microenvironment holds significant clinical importance. In recent years, multifunctional nanofibrous scaffolds have made remarkable progress in the field of infected wound treatment (Xu et al., 2025), among which electroactive scaffolds can regulate cell behavior by mimicking endogenous electrical signals (Chen et al., 2025), while natural product functionalization strategies provide a feasible pathway for achieving synergistic antibacterial, antioxidant, and immunomodulatory effects (Ali et al., 2025).

Electrospinning technology has been widely applied in tissue engineering due to its ability to fabricate nanofibrous scaffolds that mimic the structure of the native extracellular matrix (Bhardwaj and Kundu, 2010). Electrospun scaffolds possess high specific surface area, high porosity, and controllable fiber alignment, providing a favorable physical microenvironment for cell adhesion, proliferation, and migration (Azimi et al., 2020). Polycaprolactone, as an FDA-approved biodegradable polyester, exhibits good mechanical properties and biocompatibility, and is therefore a commonly used substrate for electrospun scaffolds (Komur et al., 2017). However, the inherent hydrophobicity and lack of bioactivity of PCL limit its active regulatory capacity over cellular behaviors.

In recent years, conductive biomaterials have demonstrated unique advantages in the field of tissue regeneration. Studies have shown that endogenous electrical signals play an important role in regulating cell migration, proliferation, and differentiation. It should be noted that the electroactive scaffold constructed in this study is designed to mimic and conduct the endogenous physiological electric field signals present locally in the wound, rather than applying exogenous electrical stimulation. Through this biomimetic mechanism, the electroactive scaffold provides a suitable physical microenvironment for the regulation of cellular behaviors, thereby promoting tissue repair (Funk, 2015). Carbon nanotubes (CNTs), owing to their excellent electrical conductivity, unique tubular structure, and good mechanical strength, have become ideal materials for constructing conductive biological scaffolds (Negri et al., 2020). Previous studies have confirmed that incorporating CNTs into PCL scaffolds not only endows the material with electrical conductivity but also promotes the adhesion and proliferation of fibroblasts (Suh et al., 2022).

Curcumin, a natural polyphenolic compound extracted from the rhizome of turmeric (Curcuma longa), exhibits multiple pharmacological activities, including anti-inflammatory, antioxidant, antibacterial, and pro-angiogenic effects (Anand et al., 2007). Studies have shown that curcumin can effectively scavenge excessive reactive oxygen species (ROS) at the wound site (Du et al., 2025),inhibit the expression of inflammatory cytokines (Ghandadi and Sahebkar, 2017),and exert inhibitory effects against common wound pathogens such as *Staphylococcus aureus* and *Escherichia coli*. However, the poor water solubility, rapid *in vivo* metabolism, and low bioavailability of curcumin limit its direct application in wound dressings (Luo et al., 2023).

Inspired by mussel adhesive proteins, polydopamine (PDA) coating technology provides a simple and efficient strategy for the surface functionalization of biomaterials (Yang et al., 2012). Under weakly alkaline conditions, dopamine can undergo self-polymerization to form a strongly adhesive PDA coating on various material surfaces (Yang et al., 2012). The PDA coating is rich in active functional groups such as catechol and quinone moieties, which can significantly improve the hydrophilicity of materials and serve as a secondary reaction platform for loading various bioactive molecules (Du et al., 2024). Through π–π stacking, hydrogen bonding, and Michael addition reactions, PDA can stably bind with traditional Chinese medicine molecules containing phenolic hydroxyl groups, amino groups, and other structures, thereby enabling efficient loading and sustained release of natural products such as curcumin, baicalin, and quercetin (Cui et al., 2024). Based on this property, PDA-mediated functionalization strategies with traditional Chinese medicine molecules have been applied in various tissue repair scenarios (Cui et al., 2023). For example, in bone tissue engineering, PDA coating loaded with baicalin can promote osteogenic differentiation of bone marrow mesenchymal stem cells and enhance the osseointegration capacity of titanium implants (Wang et al., 2019); in the field of nerve repair, PDA-modified scaffolds loaded with curcumin can inhibit oxidative stress and promote neuronal axon regeneration (Mao et al., 2025); in cardiovascular tissue engineering, PDA surface modification with copper-based metal-organic frameworks can improve endothelial cell function and inhibit thrombosis (Fan et al., 2019). These studies confirm that PDA-mediated loading strategies for traditional Chinese medicine molecules possess broad applicability, providing a versatile platform for constructing multifunctional biomaterials. In addition, PDA itself exhibits the ability to scavenge reactive oxygen species and can regulate macrophage polarization toward the anti-inflammatory and reparative M2 phenotype, thereby improving the wound microenvironment (Mao et al., 2025). In this study, we utilized the PDA coating to efficiently load curcumin, endowing the scaffold with multiple biological functions, including anti-inflammatory, antioxidant, and antibacterial properties.

Based on the above background, in this study we fabricated PCL/CNT conductive nanofibrous scaffolds via electrospinning technology, followed by surface modification with PDA to construct a hydrophilic active coating that serves as a bridge for efficient loading of curcumin, yielding the PCL/CNT-PDA@Cur composite functional scaffold. The morphology, structure, hydrophilicity, electrical conductivity, and curcumin loading and release behaviors of the scaffold were characterized. The effects of the scaffold on the proliferation, apoptosis, and collagen expression of L929 fibroblasts, the tube-forming capacity and angiogenesis-related gene expression of HUVECs, as well as the polarization and inflammatory cytokine regulation of RAW264.7 macrophages, were evaluated through *in vitro* experiments. Meanwhile, the antibacterial performance and underlying mechanisms against *S. aureus* and *E. coli* were assessed. Finally, the wound healing efficacy was validated in a rat full-thickness skin defect wound infection model, aiming to provide a new strategy for the treatment of complex wounds.

## 2 Materials and methods

### 2.1 Fabrication of electrospun membrane scaffolds

Electrospinning was performed at room temperature (25 °C) with a relative humidity of 35%. First, 0.01 g of carbon nanotubes (CNTs) was ultrasonically dispersed in 2 mL of hexafluoroisopropanol (HFIP), and 0.8 g of polycaprolactone (PCL) was dissolved in 8 mL of HFIP. The two solutions were then mixed to obtain the PCL/CNT spinning solution. The prepared solution was loaded into a 10 mL syringe equipped with a 21G stainless steel needle. The distance between the needle tip and the drum collector (covered with aluminum foil) was set at 20 cm, with a rotational speed of 2,000 r/min. Electrospinning was carried out at a collection voltage of −2 kV, an emitting voltage of 15 kV, and a constant flow rate of 0.02 mm/min to produce the PCL/CNT membranes.

For polydopamine coating, dopamine powder was dissolved in 10 mM Tris-HCl buffer (pH 8.5) at a concentration of 2 mg/mL. The PCL/CNT fibrous scaffolds were cut into squares of 10 mm × 10 mm and placed in a 12-well plate. Then, 1 mL of the dopamine solution was added to each well, and the plate was placed on a shaker at room temperature in the dark for 6 h. After incubation, the scaffolds were rinsed three times with sterile deionized water (5 min each wash) and freeze-dried for later use. The resulting samples were designated as PCL/CNT-PDA.

To load curcumin, 2 mg of curcumin was dissolved in 1 mL of ethanol, and the PCL/CNT-PDA scaffolds were immersed in this solution and incubated in the dark for 24 h. The scaffolds were then gently rinsed three times with sterile deionized water, freeze-dried, and stored for further use. The final samples were denoted as PCL/CNT-PDA@Cur.

### 2.2 Characterization of scaffolds

The surface morphology of the scaffolds was observed using a scanning electron microscope (SEM; JSM-7900F, JEOL, Japan). Fifty fiber diameters were randomly selected from different regions of various fibers in the SEM images, and the arithmetic mean and distribution of the diameters were measured and analyzed using ImageJ and Origin software. The elemental distribution of the scaffolds was examined by energy-dispersive X-ray spectroscopy (EDS). The microstructure of the scaffolds was observed using a transmission electron microscope (TEM; JEM-1400, JEOL, Japan).

The electrical conductivity of the scaffolds was measured using a dual-probe method with an ultra-high resistance micro-current tester (ST2643, Jingge Electronics, China). The static water contact angles of the scaffolds were determined using the sessile drop method on a contact angle/surface tension meter (JY-82C, Chengde Dingsheng, China), and the arithmetic mean values of the contact angles were analyzed using Origin software.

### 2.3 Curcumin loading and release efficiency

The concentration of curcumin in the initial curcumin solution and in the supernatant after loading was determined by measuring the absorbance at 425 nm using a UV-vis spectrophotometer. The curcumin loading efficiency was then calculated according to the standard curve and the following formula:

$$DLE\%=\frac{\left(m0-mf\right)}{m0}\times 100\%$$

where m0 is the total mass of curcumin added during the experiment, and mf is the mass of curcumin in the supernatant after centrifugation.

To evaluate the *in vitro* release profile of curcumin, absolute ethanol was used as the release medium. Scaffolds measuring 2 cm × 2 cm were placed into 15 mL light-protected centrifuge tubes, which were then placed in a thermostatic shaker incubator set at 37 °C with a shaking speed of 100 r/min. At predetermined time intervals (0.5, 1, 1.5, 2, 2.5, 3, 4, 8, 12, 24, 48, 72, 96, 120, 144, and 168 h), 1 mL of the release medium was withdrawn from each tube, and an equal volume of fresh absolute ethanol (1 mL) was added to maintain a constant total volume. The absorbance of the collected samples was measured at 425 nm using a UV-vis spectrophotometer, and the data were analyzed using Origin software.

### 2.4 Quantitative analysis of cell viability

Scaffolds from different groups were cut into pieces of 1 cm × 1 cm and immersed in 1 mL of complete medium for 24 h to obtain the corresponding extracts. L929 fibroblasts and HUVECs were seeded into 96-well plates at a density of 1 × 10^5^ cells per well, and then cultured with the extracts from the control group (without material), PCL/CNT, PCL/CNT-PDA, and PCL/CNT-PDA@Cur groups for 1, 3, and 5 days. At each time point, cell viability was assessed using a CCK-8 kit, and the optical density (OD) was measured at 450 nm using a microplate reader.

### 2.5 Fluorescence staining of cells

L929 cells were seeded into confocal culture dishes at a density of 1 × 10^6^ cells per well. After cell attachment, the cells were cultured with the scaffold extracts for 3 days. Following incubation, the cells were fixed with 4% tissue fixative and then permeabilized with 0.5% Triton X-100. Subsequently, the cells were incubated with FITC-labeled phalloidin working solution at room temperature in the dark for 30 min, followed by three washes with PBS (5 min each). The cells were then incubated with ready-to-use DAPI solution at room temperature in the dark for 10 min, and washed three times with PBS (5 min each). Finally, the cells were observed using a laser scanning confocal microscope.

HUVECs and RAW264.7 macrophages were respectively cultured with the scaffold extracts in confocal culture dishes for 3 days. After incubation, the cells were fixed with 4% tissue fixative and permeabilized with 0.5% Triton X-100. For immunostaining, RAW264.7 cells were incubated with primary antibodies against Arg-1 and iNOS, while HUVECs were incubated with primary antibody against CD31, overnight at 4 °C in the dark. After washing, the cells were incubated with the corresponding secondary antibodies in the dark. Following secondary antibody incubation, the cells were stained with ready-to-use DAPI solution at room temperature in the dark. Finally, all samples were observed under a laser scanning confocal microscope.

### 2.6 Quantitative real-time PCR

L929 fibroblasts, HUVECs, and RAW264.7 macrophages were cultured in the scaffold extracts for 3 days to evaluate the effects on the expression of collagen type I and type III in L929 fibroblasts, VEGF and CD31 in HUVECs, and TNF-α and Arg-1 in RAW264.7 cells. Total RNA was extracted using a rapid RNA extraction kit (Accurate Biology, China). The purity and concentration of total RNA were determined using a microspectrophotometer (NanoDrop One). Reverse transcription of total RNA to cDNA was performed using the RT Master Mix for qPCR II kit (MCE, NJ, United States). qPCR was carried out using the SLAN-96S Real-Time PCR System (Hongshi Medical Technology Co., Ltd., Shanghai, China) with SYBR Green qPCR Master Mix (MCE, NJ, United States).

**Table:** 

Primers	Sequence (5′ to 3′)F	Sequence (5′ to 3′)R
Col I	GCT​CCT​CTT​AGG​GGC​CAC​T	CACGTCTCACCATTGGGG
Col III	CTG​TAA​CAT​GGA​AAC​TGG​GGA​AA	CCA​TAG​CTG​AAC​TGA​AAA​CCA​CC
VEGF	AGGGCAGAATCATCACGA	CACACAGGATGGCTTGAA
CD31	GAG​TCC​TGC​TGA​CCC​TTC​TG	CAC​TCC​TTC​CAC​CAA​CAC​CT
TNF-α	GGC​AGG​TTC​TGT​CCC​TTT​CA	TCT​TCT​GCC​AGT​TCC​ACG​TC
ARG1	TTT​CTC​AAA​AGG​ACA​GCC​TCG	ACA​GAC​CGT​GGG​TTC​TTC​AC

### 2.7 Apoptosis staining

L929 cells were seeded into 6-well plates at a density of 1 × 10^6^ cells per well. After cell attachment, the cells were cultured with the scaffold extracts for 3 days. Following incubation, apoptosis was assessed using the Hoechst 33258 staining kit (Beyotime, China) according to the manufacturer’s instructions. After staining, the cells were observed under a fluorescence microscope with excitation/emission filters at 364/454 nm to evaluate cell apoptosis in each group.

### 2.8 *In vitro* angiogenesis assay of HUVECs

HUVECs were cultured with the scaffold extracts for 3 days. After culture, the cells were trypsinized and resuspended to obtain a single-cell suspension at a density of 4 × 10^5^ cells/mL. A 96-well plate was pre-coated with Matrigel and allowed to solidify at 37 °C for 30 min. The prepared HUVEC suspension was then seeded onto the Matrigel-coated wells and incubated for 12 h at 37 °C in a 5% CO_2_ incubator. After incubation, tube formation was observed under an inverted microscope, and images were captured for subsequent quantitative analysis.

### 2.9 Quantitative analysis of cell viability under H_2_O_2_-induced oxidative stress

To determine whether the scaffolds possess antioxidant properties and can protect cells against reactive oxygen species (ROS)-induced damage, the CCK-8 assay was employed. L929 fibroblasts, HUVECs, and RAW264.7 macrophages were seeded into 6-well plates at a density of 1 × 10^6^ cells per well. After 24 h of incubation, the culture medium was removed. Cells in the experimental groups were cultured with scaffold extracts, while those in the control group were cultured with normal medium for 3 days. Following incubation, the cells were exposed to H_2_O_2_ solution at a concentration of 100 μM and incubated at 37 °C in the dark for 5 h. Cell viability was then assessed using the CCK-8 kit according to the manufacturer’s instructions.

### 2.10 Intracellular ROS detection under H_2_O_2_ stimulation

L929 fibroblasts, HUVECs, and RAW264.7 macrophages were seeded into 6-well plates. The experimental groups were cultured with scaffold extracts, while the control group was cultured with normal medium for 3 days. After incubation, the cells were treated with 500 μL of 50 μM H_2_O_2_ per well and further incubated for 2 h, followed by two washes with PBS to remove residual H_2_O_2_.

Subsequently, the cells were incubated with DCFH-DA fluorescent probe (diluted with PBS to a final concentration of 10 μM), with at least 1 mL added to each well of the 6-well plate. After incubation at 37 °C in the dark for 60 min, the cells were washed with PBS. Finally, the intracellular ROS levels were observed using a laser scanning confocal microscope with FITC excitation/emission filters (Ex/Em = 496/516 nm) to visualize the cells on the surface of the fibrous membranes in each group.

### 2.11 Antibacterial assay

*Staphylococcus aureus* (*Staphylococcus aureus*, ATCC 6538) and *E. coli* (*Escherichia coli*, ATCC 25922) were used as model bacterial strains. The bacteria were separately inoculated onto Luria–Bertani (LB) solid agar plates (Biosharp, China) and incubated at 37 °C. Prior to the experiments, single colonies were transferred to LB liquid medium (Biosharp, China) and cultured overnight to reach the logarithmic growth phase. Bacterial suspensions in the logarithmic phase were used for subsequent experiments.

### 2.12 *In vitro* antibacterial evaluation

To evaluate the bactericidal efficacy of the scaffolds against *S. aureus* and *E. coli* that had adhered to and proliferated on the scaffold surface, 500 μL of bacterial suspension (1.0 × 10^6^ CFU/mL) was added to each well of a 24-well plate containing the scaffolds, followed by co-incubation at 37 °C for 12 h. After incubation, the scaffolds were rinsed three times with sterile PBS, transferred to fresh plates, and immersed in 1 mL of PBS with ultrasonication for 1 min to detach the adherent bacteria. The resulting bacterial suspension was diluted 1000-fold, and 10 μL of the diluted suspension was spread onto LB solid agar plates (Biosharp, China). After incubation at 37 °C for 12 h, the colonies were counted. The bacterial survival rate was calculated using the following formula:

$$\text{Bacterial survival rate }\left(\%\right)=\text{Ne}/\text{Nc}\times 100$$

where N_e_ represents the number of colonies in the experimental group and N_c_ represents the number of colonies in the control group.

To investigate the antibacterial mechanism of the scaffolds, the interaction between the scaffolds and bacteria (*S. aureus* and *E. coli*), as well as the morphological changes of bacterial cell membranes, were observed using scanning electron microscopy (SEM). Briefly, 500 μL of bacterial suspension (1.0 × 10^6^ CFU/mL) was co-incubated with the scaffolds in a 24-well plate at 37 °C for 12 h. After incubation, the scaffolds were gently rinsed three times with sterile PBS, fixed with 2.5% glutaraldehyde fixative solution (Solarbio, China) overnight at 4 °C, and then washed with PBS. Subsequently, the samples were dehydrated through a graded series of ethanol solutions (30, 50, 70, 90, 95, and 100% (v/v)), with 15 min per step. After natural drying, the samples were sputter-coated with gold and observed under SEM to examine bacterial morphology on the scaffold surfaces.

### 2.13 Biofilm eradication assay

To evaluate the inhibitory effect of the scaffolds on bacterial biofilm formation, 100 µL of *S. aureus* suspension (1.0 × 10^6^ CFU/mL) in culture medium was added to each well of a 96-well plate and incubated at 37 °C for 48 h, with the medium replaced every 24 h. After incubation, the culture medium was removed, and the non-adherent bacteria were gently washed away three times with 200 µL of sterile PBS. The biofilms formed on the well surfaces were then harvested for further experiments.

The scaffolds from each group were added to the bacterial biofilms in PBS and incubated at 37 °C with continuous shaking for 6 h. A group without any scaffold served as the blank control. After incubation, the scaffolds were removed, and the wells were rinsed three times with PBS to remove floating bacteria. Subsequently, 200 µL of absolute ethanol was added to fix the biofilms at 4 °C for 15 min. After removing the ethanol, the biofilms were stained with 300 µL of 1% (w/v) crystal violet solution for 15 min.

Following staining, the wells were washed three times with PBS to remove excess crystal violet, and photographs were taken. The plates were then dried at 37 °C. Finally, 500 µL of absolute ethanol was added to each well and incubated for 30 min to dissolve the crystal violet (or alternatively, after complete drying, 100 µL of 33% (v/v) glacial acetic acid was added to each well and incubated at 37 °C for 30 min). The absorbance of the resulting solution was measured at 450 nm using a microplate reader. Each experiment was repeated four times independently.

### 2.14 Bacterial membrane permeability

To evaluate the effect of the scaffolds on the cell membrane permeability of *S. aureus*, the ONPG (o-nitrophenyl-β-D-galactopyranoside) assay was employed. Briefly, 500 μL of bacterial suspension (1.0 × 10^6^ CFU/mL) was co-incubated with the scaffolds in a 24-well plate at 37 °C for 6 h. After incubation, the bacteria adhering to the scaffold surface were immersed in 500 μL of ONPG solution (prepared in NaH_2_PO_4_ buffer, pH 7.0). The absorbance of the supernatant was then recorded at 405 nm using a spectrophotometric microplate reader.

### 2.15 Bacterial DNA damage response (SOS response)

To evaluate the effect of the scaffolds on the expression of bacterial SOS response-related genes, 500 μL of bacterial suspension (1.0 × 10^6^ CFU/mL) was co-incubated with the scaffolds at 37 °C for 6 h. After incubation, the bacteria were collected, and total RNA was extracted using an RNAprep Pure Cell/Bacteria Kit. The extracted RNA was then reverse-transcribed into cDNA using the RT Master Mix for qPCR II kit (MCE, NJ, United States). Subsequently, qPCR was performed using the SLAN-96S Real-Time PCR System (Hongshi Medical Technology Co., Ltd., Shanghai, China) with SYBR Green qPCR Master Mix (MCE, NJ, United States) to detect the expression levels of SulA, RecA, and LexA. The relative expression levels were calculated using the ^2−ΔΔCt^ method, with 16S rRNA used as the internal reference gene for normalization.

**Table:** 

Primers	Sequence (5′ to 3′)F	Sequence (5′ to 3′)R
sulA	GGWTACCTTGTTACGACTT	GCC​TGA​AGT​GAG​CTC​AAT​CAA​TCC
recA	TCC​GGT​AAA​ACC​ACG​CTG​AC	CGT​GCG​TAG​ATT​GGG​TCC​AG
lexA	CGC​GGC​TGA​AGA​ACA​TCT​GA	GCG​GCA​ACC​CTT​CTT​CCT​CT
16srRNA	CGGTGAATACGTTCYCGG	GGWTACCTTGTTACGACTT

### 2.16 Animal model and surgical procedure

To evaluate the effects of different scaffold materials on skin regeneration, a rat full-thickness skin defect infection model was established. All animal procedures were approved by the Animal Ethics Committee of Peking University People`s Hospital (Approval No. 2026PHE089). Forty female Sprague-Dawley (SD) rats, each weighing 180–200 g, were randomly divided into four groups: the control group (untreated wound), PCL/CNT group, PCL/CNT-PDA group, and PCL/CNT-PDA@Cur group.

All rats were anesthetized by inhalation of isoflurane (induction at 3%, maintenance at 1.5%; Ward Life Science Technology Co., Ltd., Shenzhen, China). Under sterile conditions, a circular full-thickness skin defect with a diameter of 10 mm was created on the dorsum of each rat using a skin punch biopsy tool. Subsequently, each wound was covered with a corresponding scaffold measuring 15 mm × 15 mm. Wound healing was monitored by taking photographic images on postoperative days 0, 3, 7, and 14. The wound areas were measured and analyzed using ImageJ software, and the wound closure rate was calculated accordingly.

$$Wound\;closure\;rate\left(\%\right)=\frac{Initial\;wound\;area-Actual\;wound\;area}{Initial\;wound\;area}\times 100$$

### 2.17 Histological analysis

On postoperative days 3, 7, and 14, Rats were euthanized using a 50% volume per minute displacement rate of carbon dioxide for 10.5 min to ensure irreversible euthanasia, and full-thickness skin tissues containing the wound area were excised. All tissue samples were immediately fixed in 4% paraformaldehyde at 4 °C for 24 h. After fixation, the tissues were processed through a standard series of dehydration, embedded in paraffin, and sectioned into 5-μm-thick slices for subsequent histological and immunofluorescence analyses.

To evaluate wound healing, collagen deposition, and angiogenesis, tissue sections were stained with hematoxylin and eosin (H&E) and Masson`s trichrome, respectively. For specific visualization of newly formed blood vessels, sections were subjected to immunofluorescence staining using a primary antibody against CD31 (dilution 1:500).

To evaluate the inflammatory regulation and reparative processes within the wound microenvironment, additional sections were subjected to multiplex immunofluorescence staining. Macrophages were labeled using primary antibodies against CD86 (ab119857, Abcam, United States) for the M1 phenotype and CD206 (ab8918, Abcam, United States) for the M2 phenotype. The pro-inflammatory cytokine TNF-α and anti-inflammatory cytokine IL-10 were detected using primary antibodies against TNF-α and IL-10 (both at a dilution of 1:200). Following primary antibody incubation, the sections were incubated with the corresponding Alexa Fluor 488- or Alexa Fluor 555-conjugated goat anti-rabbit/mouse secondary antibodies (A-11034/A-21422, Molecular Probes, United States) for visualization and observation.

### 2.18 Statistical analysis

All data are presented as mean ± standard deviation (SD). All *in vitro* experiments were independently repeated at least 3 times (n ≥ 3); each group in the animal experiments consisted of 10 rats (n = 10); and for immunofluorescence and ELISA quantitative analyses, each group consisted of 5 rats (n = 5). Statistical analyses were performed using SPSS 13.0 software. Comparisons between two groups were analyzed using Student’s t-test, while comparisons among three or more groups were evaluated by one-way analysis of variance (ANOVA). Statistical significance was defined as *p < 0.05, **p < 0.01, and ***p < 0.001, and ns indicates no significant difference (p* > 0.05).

## 3 Results and discussion

Skin wound healing is a highly coordinated biological process involving the sequential occurrence of multiple stages, including inflammation, cell proliferation, angiogenesis, and tissue remodeling. However, under complex pathological conditions, the wound microenvironment is often characterized by persistent inflammation, excessive accumulation of reactive oxygen species (ROS), bacterial infection, and impaired angiogenesis, which collectively impede the healing process. Therefore, the development of functional wound dressings capable of simultaneously modulating the aforementioned multiple pathological factors holds significant clinical importance. In this study, by combining electrospinning technology with polydopamine-mediated surface functionalization, we successfully constructed a curcumin-loaded electroactive nanofibrous scaffold and systematically evaluated its application potential in wound repair from multiple dimensions.

### 3.1 Fabrication and physicochemical characterization of scaffolds

A fundamental prerequisite for constructing an ideal wound dressing is its ability to mimic the structure of the native extracellular matrix (ECM) and provide a favorable physical microenvironment. In this study, PCL/CNT composite nanofibrous scaffolds were successfully fabricated via electrospinning technology, and further modified through polydopamine (PDA) surface coating and curcumin loading to obtain the multifunctional electroactive wound dressing PCL/CNT-PDA@Cur.

Scanning electron microscopy (SEM) observations (Figure 1A) revealed that all fibrous scaffolds exhibited a randomly distributed, uniform fibrous network without bead-like defects. This structure, characterized by high specific surface area and high porosity, is favorable for cell adhesion, spreading, and mass exchange, thereby providing a conducive physical microenvironment for cell proliferation and ultimately accelerating wound closure. Notably, compared with the unmodified PCL/CNT scaffolds, the PDA-modified PCL/CNT-PDA and PCL/CNT-PDA@Cur scaffolds displayed slightly rougher fiber surfaces, which could be attributed to the successful deposition of the PDA coating.

**FIGURE 1:**
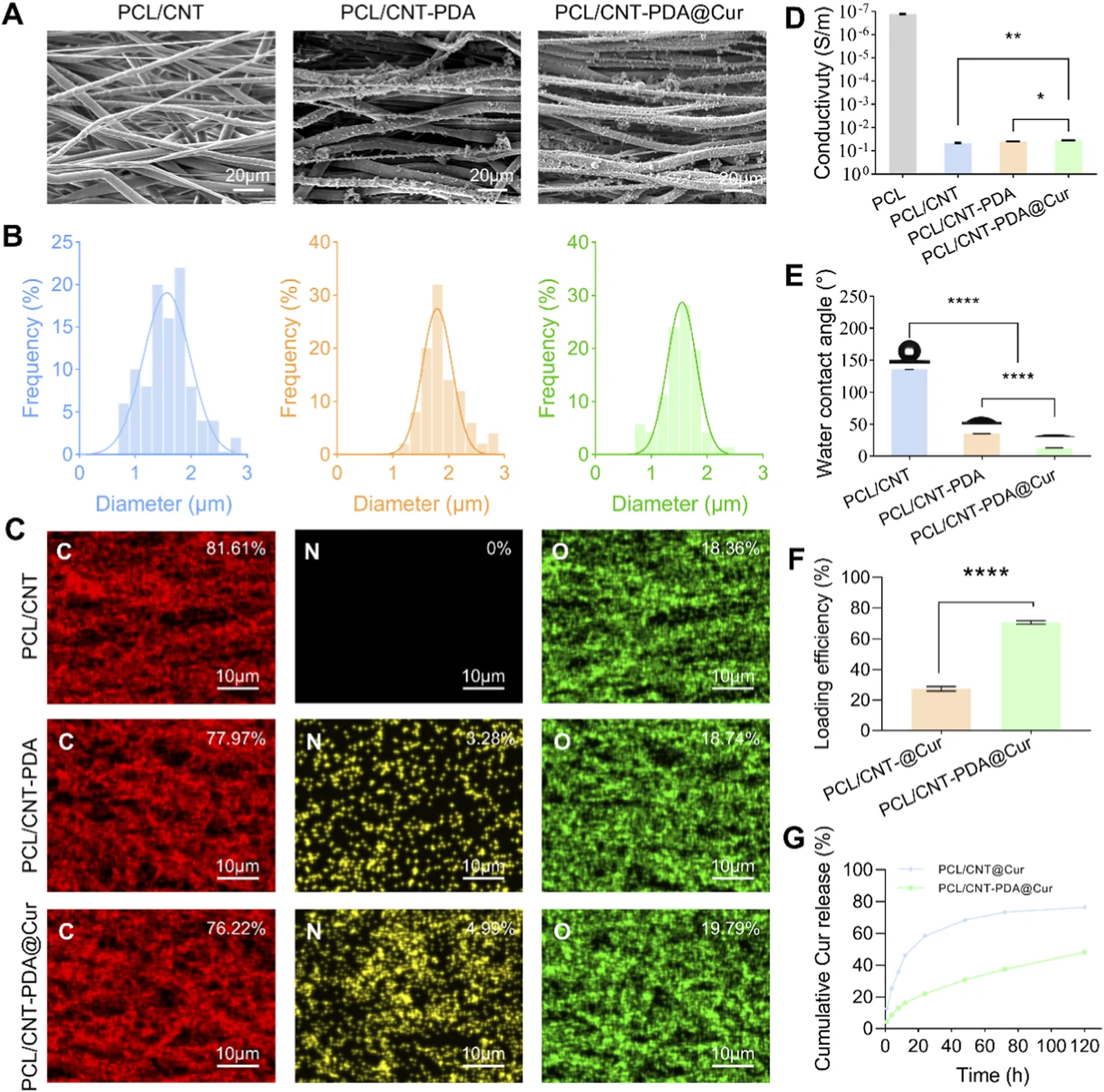
**(A)** SEM images of the fibrous scaffolds; **(B)** Fiber diameter distribution; **(C)** EDS elemental mapping; **(D)** Electrical conductivity (n = 3); **(E)** Water contact angle (n = 3); **(F)** Curcumin loading efficiency of PCL/CNT@Cur and PCL/CNT-PDA@Cur scaffolds (n = 3); **(G)** Cumulative curcumin release profiles from PCL/CNT@Cur and PCL/CNT-PDA@Cur scaffolds (n = 3). All data are presented as mean ± SD, and statistical significance was determined by one-way ANOVA followed by Tukey’s *post hoc* test; *p < 0.05, **p < 0.01, ***p < 0.001.

Fiber diameter distribution analysis (Figure 1B) showed a slight increasing trend in the average fiber diameter after PDA coating and curcumin loading; however, the overall diameter remained at the submicron scale, ensuring good flexibility and breathability of the scaffolds. In this study, SEM observations confirmed the fibrous structure of the scaffolds, and the fiber morphology was well maintained after PDA modification and curcumin loading, indicating that the layer-by-layer modification did not compromise the microscale architecture of the scaffolds.

Surface chemistry is a critical factor influencing the biological response of biomaterials. Energy-dispersive X-ray spectroscopy (EDS) analysis (Figure 1C) confirmed the successful fabrication of the scaffolds. The results showed that the PCL/CNT scaffolds were mainly composed of C and O elements, whereas N element signals were detected in the PCL/CNT-PDA and PCL/CNT-PDA@Cur groups, clearly indicating the presence of the PDA coating, while the distribution of C and O elements corresponded to the components of PCL and CNTs. In addition to the N element, the O content slightly increased on the surface of the PCL/CNT-PDA@Cur scaffolds, consistent with the successful loading of curcumin.

Furthermore, the incorporation of CNTs endowed the scaffolds with stable electrical conductivity. Endogenous electrical signals play an important role in the wound healing process by regulating cell migration, proliferation, and differentiation (Wang and Zhao, 2010). The electrical conductivity measurements (Figure 1D) revealed that the PCL/CNT scaffolds exhibited a conductivity on the order of 10^−2^ S/m, indicating that the introduction of CNTs successfully established a conductive network. After PDA coating modification, the conductivity of the PCL/CNT-PDA scaffolds remained at the level of 10^−2^ S/m, suggesting that PDA modification did not significantly disrupt the conductive pathways formed by the CNTs. This conductivity level falls within the range of biological tissue conductivity and is sufficient to support the conduction of endogenous electrical signals between cells without the need for an external power source. Therefore, the electroactive function of this scaffold is designed to mimic the physiological microenvironment and regulate cell behavior by conducting endogenous electrical signals to promote tissue repair.

PDA surface modification served as a key strategy for the functionalization of the scaffolds. As a mussel-inspired biomimetic material, PDA can form strongly adhesive coatings on various substrates, and its abundant catechol and amino groups endow the material with multiple functions. Water contact angle measurements (Figure 1E) showed that the PCL/CNT scaffolds exhibited a static water contact angle of 135.46° ± 0.19°, indicating pronounced hydrophobicity. After PDA modification, the contact angle of the PCL/CNT-PDA scaffolds significantly decreased to 35.30° ± 0.12° (P < 0.01), reflecting a marked improvement in hydrophilicity. This result is consistent with previous reports, as the abundant catechol and amino groups in the PDA coating effectively enhance the surface hydrophilicity of materials. After curcumin loading, the contact angle of the PCL/CNT-PDA@Cur scaffolds slightly increased but remained at a relatively low level, indicating that the hydrophilicity improvement conferred by PDA modification was maintained after drug loading. Good hydrophilicity not only facilitates cell adhesion and spreading but also promotes macrophage polarization toward the anti-inflammatory M2 phenotype (Lv et al., 2018).

### 3.2 Curcumin loading and release profiles

Curcumin, a natural polyphenolic compound, possesses favorable anti-inflammatory, antioxidant, and antibacterial activities. However, its poor water solubility and low photostability limit its direct application in wound dressings. To evaluate the enhancing effect of PDA coating on curcumin loading, we also prepared a control sample without PDA modification but directly loaded with curcumin, designated as PCL/CNT@Cur. Ultraviolet-visible spectrophotometry results (Figure 1F) revealed that the curcumin loading efficiency of the PCL/CNT-PDA scaffolds reached 71.38% ± 1.04%, which was significantly higher than that of the unmodified control group (27.84% ± 0.94%, P < 0.01). This result indicates that the abundant catechol groups in the PDA coating can interact with curcumin molecules through π–π stacking interactions, thereby enabling efficient drug loading.

The *in vitro* release profiles (Figure 1G) showed that the unmodified PCL/CNT scaffolds released more than 60% of the loaded curcumin within 24 h, exhibiting a pronounced burst release. In contrast, the PCL/CNT-PDA@Cur scaffolds displayed a much more sustained and controlled release behavior: approximately 25% of the drug was released within the initial 24 h, followed by a gradual stabilization of the release rate, reaching a cumulative release of about 40% at 72 h, after which the release continued at a slow rate. Given that the inflammatory phase of skin wound healing typically lasts 48–72 h, the PCL/CNT-PDA@Cur scaffolds, which continuously release curcumin during this critical time window, can effectively exert their anti-inflammatory and antioxidant functions, while the subsequent slow release contributes to maintaining the stability of the local microenvironment.

The sustained release behavior achieved by PDA modification can be primarily attributed to the multiple interactions between the PDA layer and curcumin, including π–π stacking, hydrogen bonding, and possible covalent interactions, which collectively retard the diffusion of curcumin into the release medium. However, this sustained-release system also has inherent limitations. As shown in Figure 1G, the cumulative release rate of the PCL/CNT-PDA@Cur scaffold over an extended period was significantly lower than that of the unmodified PCL/CNT control group. This is because the π–π stacking and hydrogen bonding interactions between PDA and curcumin, while delaying drug diffusion, also restrict the total amount of free drug released. This characteristic suggests that in future applications, it may be necessary to appropriately increase the initial drug loading to ensure that the local concentration of free curcumin in the wound reaches effective therapeutic levels. Furthermore, strategies such as adjusting the PDA coating thickness, optimizing the drug loading concentration, or introducing degradable crosslinkers into the PDA coating may help achieve an improved balance between “burst-release suppression” and “release efficiency.”

In addition, the use of absolute ethanol as the release medium in this study was dictated by the extremely low water solubility of curcumin. Although this method is a common *in vitro* evaluation approach for curcumin sustained-release systems, it differs from the aqueous physiological environment of wounds. Future studies may explore release testing in water/ethanol mixed systems or protein-containing simulated body fluids to more closely approximate *in vivo* conditions.

### 3.3 *In vitro* regulation of cell behavior by the scaffolds

An ideal wound dressing must possess favorable cytocompatibility and actively promote the functions of tissue repair-related cells (Shen et al., 2022). Curcumin, as a natural polyphenolic compound, endows the scaffold with multiple biological functions. To evaluate the effects of the scaffolds on key reparative cells, L929 fibroblasts, HUVECs, and RAW264.7 macrophages were employed as cell models to investigate cellular responses to different scaffold treatments.

HUVECs and L929 fibroblasts are the primary effector cells during wound healing, and their proliferation capacity directly influences granulation tissue formation, wound closure, and tissue repair (Rai et al., 2023). The CCK-8 cell viability assay revealed that none of the PCL/CNT, PCL/CNT-PDA, or PCL/CNT-PDA@Cur scaffolds exhibited cytotoxicity. After 1, 3, and 5 days of culture with the scaffolds, both HUVECs and L929 cells showed a time-dependent increase in cell viability. Notably, the PCL/CNT-PDA@Cur group exhibited the highest cell viability at days 3 and 5 for both HUVECs (Supplementary Figure S1) and L929 cells (Supplementary Figure S2), significantly promoting the proliferation of both cell types.

Cytoskeleton staining results (Figure 2A) showed that after 3 days of culture, L929 cells in the PCL/CNT-PDA@Cur group displayed the most extended morphology, with a significantly larger spreading area compared with the other groups, confirming the highest cell migration rate in this group. Quantitative analysis of F-actin fluorescence intensity further validated this difference (Figure 2F). Cells formed close interactions with the fibers, with cell pseudopodia extending along the fiber orientation. qPCR analysis (Figures 2G,H) revealed that the mRNA expression levels of collagen type I (Col I) and collagen type III (Col III) in L929 cells were significantly higher in the PCL/CNT-PDA@Cur group than in the other groups (*P* < 0.01), indicating that this scaffold effectively promoted Col I and Col III production and extracellular matrix synthesis by fibroblasts, and further calculation of the Col I/Col III mRNA ratio showed an increase in this group compared to the PCL/CNT group, suggesting that the collagen type ratio tended toward a more mature repair state, thereby activating fibroblast function and facilitating wound closure and repair (Hong et al., 2023).

**FIGURE 2:**
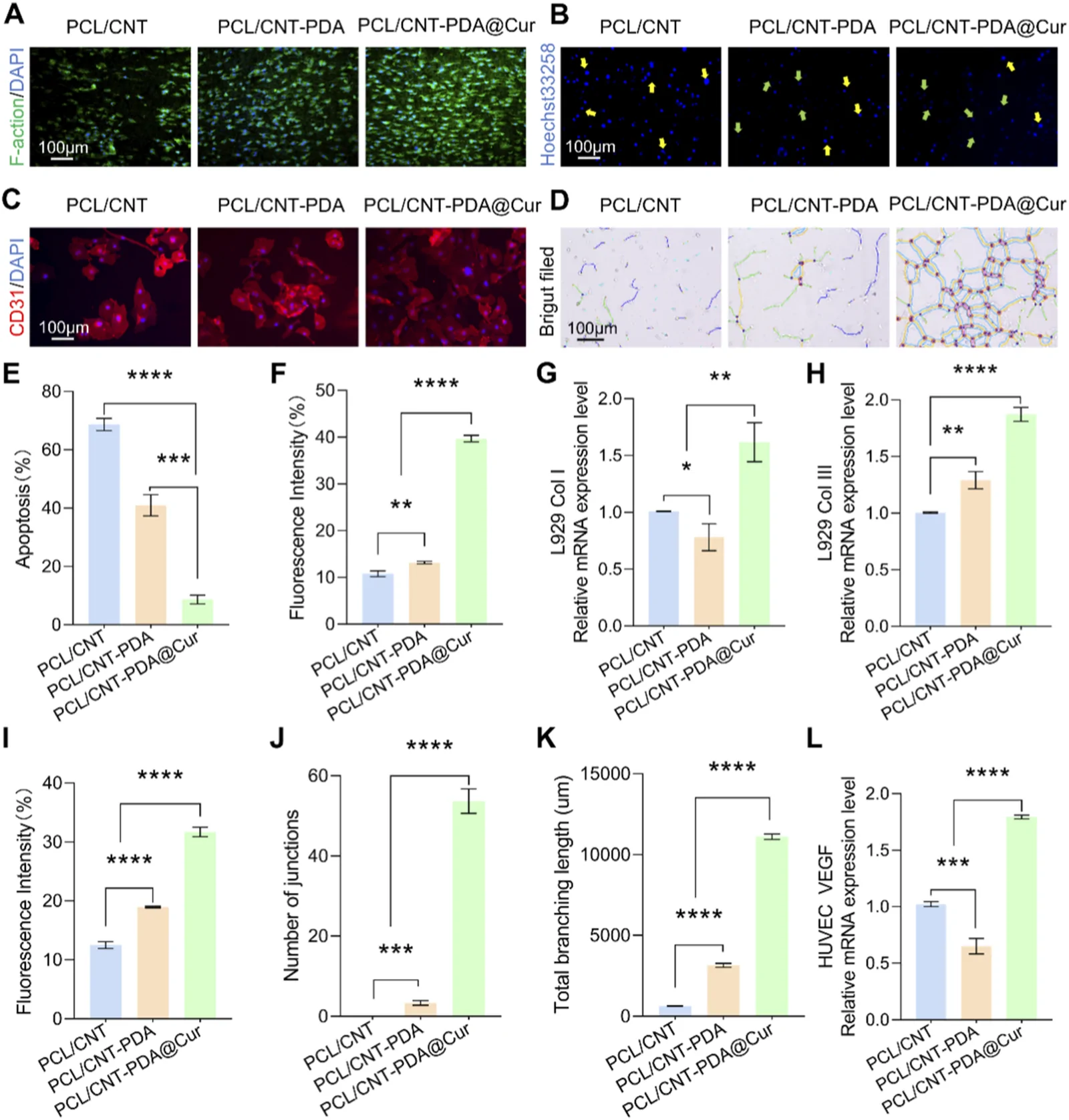
**(A)** Phalloidin staining of the cytoskeleton in L929 cells after 3 days of culture; **(B)** Hoechst 33258 staining of L929 cells cultured with different scaffolds under H_2_O_2_ stimulation (green arrows: viable cells; yellow arrows: apoptotic cells); **(C)** Immunofluorescence staining of CD31 (red) and DAPI (blue) in HUVECs after 3 days of culture; **(D)** Tube formation of HUVECs cultured *in vitro*; **(E)** Apoptosis rate (n = 3); **(F)** Fluorescence intensity of phalloidin (n = 3); Relative mRNA expression levels of Col I **(G)** and Col III **(H)** (n = 3); **(I)** Fluorescence intensity of CD31 (n = 3); Quantitative analysis of **(J)** the number of junctions and **(K)** total branching length in the HUVEC network structures under different treatment conditions (n = 3); **(L)** Relative mRNA expression level of VEGF (n = 3). All data are presented as mean ± SD, and statistical significance was determined by one-way ANOVA followed by Tukey’s *post hoc* test; *p < 0.05, **p < 0.01, ***p < 0.001.

Furthermore, the regulatory effects of the scaffolds on HUVECs were equally remarkable. Immunofluorescence staining results (Figure 2C) showed that HUVECs cultured on the PCL/CNT-PDA@Cur scaffolds for 3 days exhibited the highest CD31 expression level, and quantitative fluorescence intensity analysis (Figure 2I) confirmed that it was significantly higher than that in the PCL/CNT and PCL/CNT-PDA groups (*P* < 0.01). As CD31 is a key marker of endothelial cell activation and angiogenesis (Dhumale et al., 2023), its upregulated expression suggests that the PCL/CNT-PDA@Cur scaffold effectively promotes endothelial cell functional activation. The *in vitro* tube formation assay further confirmed the pro-angiogenic potential of this scaffold (Figures 2D,J,K). HUVECs in the PCL/CNT-PDA@Cur group formed the most well-developed tubular network structures, with both the number of junctions and total branching length significantly higher than those in the other groups (*P* < 0.01). This observation indicates that the synergistic action of curcumin and PDA on the scaffold surface provides a favorable microenvironment for endothelial cell adhesion and migration, thereby promoting the formation of vascular-like structures. qPCR analysis further demonstrated (Figure 2L) that the mRNA expression level of VEGF was significantly upregulated in the PCL/CNT-PDA@Cur group, suggesting that this scaffold may promote angiogenesis through activation of the VEGF signaling pathway. VEGF is a master regulator of angiogenesis, and its upregulation is closely associated with endothelial cell proliferation, migration, and lumen formation (Wang R. et al., 2025). Collectively, these results indicate that the PCL/CNT-PDA@Cur scaffold, through synergistic regulation of endothelial cell function and angiogenesis-related gene expression, provides favorable conditions for neovascularization during the wound healing process.

### 3.4 Antioxidant and immunomodulatory properties of the scaffolds

Antioxidant and immunomodulatory functions represent another core attribute of the PCL/CNT-PDA@Cur scaffolds. Chronic non-healing wounds are often accompanied by excessive accumulation of reactive oxygen species (ROS), leading to oxidative stress damage and persistent inflammation. To evaluate the antioxidant performance of the scaffolds, systematic assessments were conducted through DPPH free radical scavenging assays and intracellular ROS detection. The DPPH assay results (Figure 3D) showed that the PCL/CNT-PDA@Cur scaffolds exhibited a significantly higher free radical scavenging rate than the PCL/CNT scaffolds (*P* < 0.01), indicating excellent free radical scavenging capacity. This effect can be primarily attributed to the synergistic antioxidant action of the reducing groups in PDA and curcumin, which together endow the scaffold with robust antioxidant properties.

**FIGURE 3:**
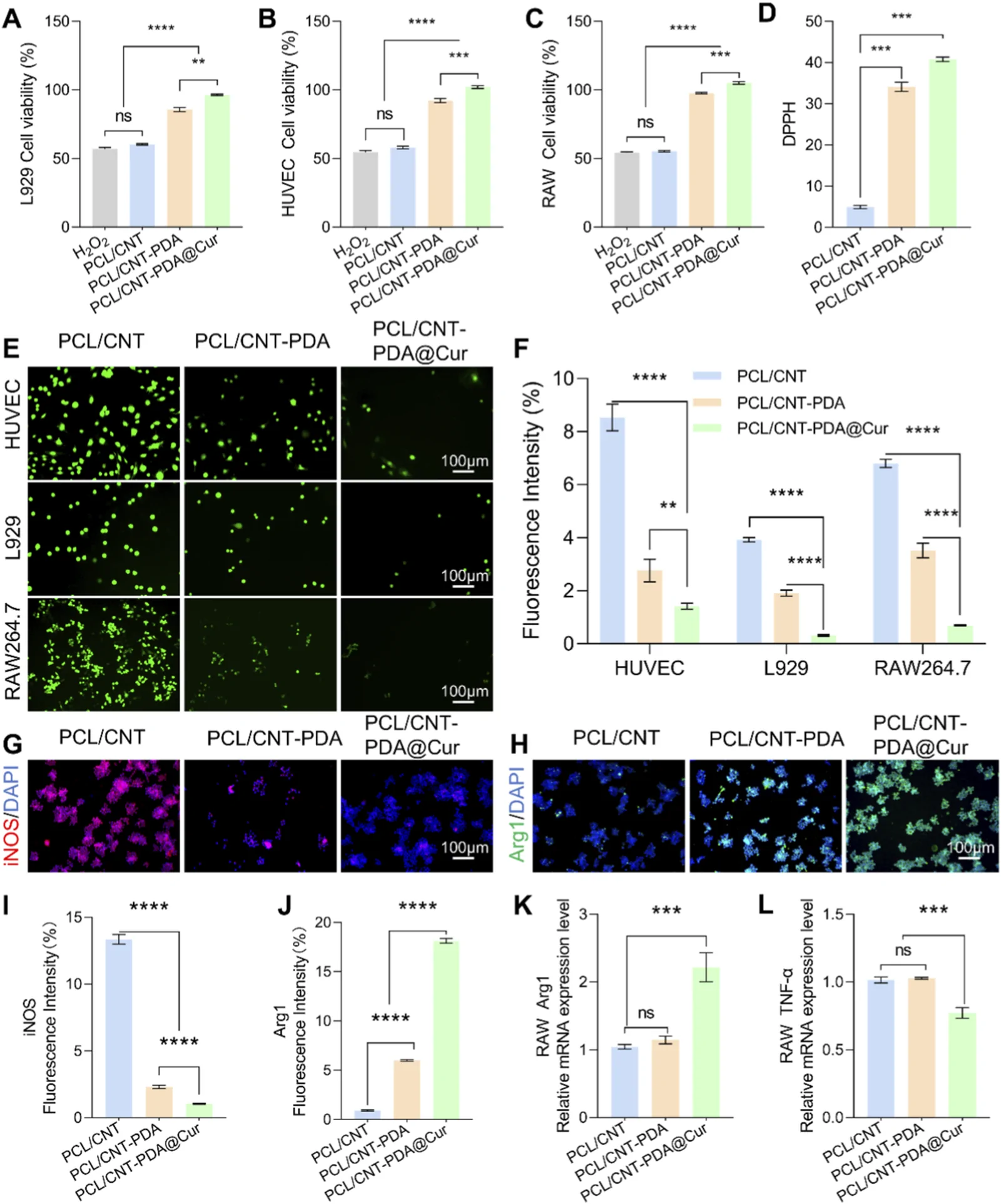
**(A–C)** Cell viability of L929, HUVEC, and RAW264.7 cells under H_2_O_2_ exposure (n = 3); **(D)** DPPH free radical scavenging capacity (n = 3); **(E)** Fluorescence staining of intracellular ROS in L929, HUVEC, and RAW264.7 cells under H_2_O_2_ stimulation and **(F)** quantitative analysis of fluorescence intensity (n = 3); **(G, H)** Immunofluorescence staining of iNOS **(G)** and Arg-1 **(H)** in RAW264.7 macrophages under inflammatory stimulation, and **(I, J)** quantitative analysis of fluorescence intensity for iNOS **(I)** and Arg-1 **(J)** (n = 3); **(K, L)** Relative mRNA expression levels of Arg-1 **(K)** and TNF-α **(L)** in RAW264.7 cells (n = 3). All data are presented as mean ± SD, and statistical significance was determined by one-way ANOVA followed by Tukey’s *post hoc* test; *p < 0.05, **p < 0.01, ***p < 0.001.

Under H_2_O_2_-induced oxidative stress conditions, CCK-8 assay results (Figures 3A–C) demonstrated that the PCL/CNT-PDA@Cur scaffolds significantly improved the viability of L929, HUVEC, and RAW264.7 cells, with statistically significant differences compared with the PCL/CNT group (*P* < 0.01). DCFH-DA fluorescence probe detection (Figures 3E,F) revealed that intracellular ROS levels in the PCL/CNT-PDA@Cur group were significantly lower than those in the PCL/CNT group (*P* < 0.01), indicating that this scaffold effectively reduced intracellular ROS levels and scavenged excessive ROS generated under oxidative stress.

Hoechst 33258 staining results (Figures 2B,E) showed that under H_2_O_2_ stimulation, L929 cells in the PCL/CNT group exhibited obvious apoptotic features, including nuclear condensation and fragmentation, whereas cells in the PCL/CNT-PDA@Cur group maintained essentially normal nuclear morphology, with a significantly reduced apoptosis rate (*P* < 0.01). This finding confirms that the scaffold effectively protects cells from oxidative stress-induced apoptosis.

To evaluate the immunomodulatory properties of the scaffolds, the phenotypic changes of RAW264.7 macrophages were analyzed by immunofluorescence staining and qPCR. Immunofluorescence results (Figures 3G–J) revealed that under inflammatory stimulation induced by lipopolysaccharide (LPS) and interferon-γ (IFN-γ), the expression of the M1 macrophage marker iNOS was significantly decreased in the PCL/CNT-PDA@Cur group, with progressively diminishing fluorescence intensity, while the expression of the M2 marker Arg-1 was significantly increased, showing an opposite fluorescence trend. These results indicate that this scaffold effectively regulates macrophage polarization, with statistically significant differences compared with the PCL/CNT group (*P* < 0.01). qPCR analysis further confirmed (Figures 3K,L) that in the PCL/CNT-PDA@Cur group, the mRNA expression level of the pro-inflammatory cytokine TNF-α was significantly downregulated, whereas the mRNA expression level of the anti-inflammatory marker Arg-1 was significantly upregulated (*P* < 0.01). Collectively, these results demonstrate that the PCL/CNT-PDA@Cur scaffold possesses potent immunomodulatory potential, effectively promoting macrophage polarization toward the anti-inflammatory and reparative M2 phenotype. The transition of macrophages from the pro-inflammatory M1 phenotype to the anti-inflammatory and reparative M2 phenotype is a critical step in the shift from the inflammatory phase to the proliferative phase of wound healing (Wang H. et al., 2025). In this study, the dual regulatory effects of PDA and curcumin provided favorable conditions for this transition, contributing to the overall improvement of the wound microenvironment.

### 3.5 Antibacterial activity and underlying mechanisms

Antibacterial performance is a critical functional indicator of wound dressings (Wang et al., 2026). *Staphylococcus aureus* and *Escherichia coli* are the most common pathogens in wound infections. Given that wound infection is a major factor impeding healing, antibacterial function is of great significance for promoting skin defect repair. In this study, the antibacterial performance of the PCL/CNT-PDA@Cur scaffolds against *S. aureus* and *E. coli* was systematically evaluated.

Plate colony counting results (Figures 4A,C,D) demonstrated that the PCL/CNT-PDA@Cur scaffolds achieved bactericidal rates exceeding 90% against both *S. aureus* and *E. coli*, which were significantly higher than those of the PCL/CNT scaffolds (*P* < 0.01). SEM observations (Figure 4B) revealed that after co-culture with the PCL/CNT-PDA@Cur scaffolds, both bacterial species exhibited pronounced morphological changes, including shrinkage, rupture, and leakage of intracellular contents (indicated by yellow arrows), suggesting disruption of bacterial membrane integrity. In contrast, bacteria in the control group maintained intact morphology with smooth surfaces.

**FIGURE 4:**
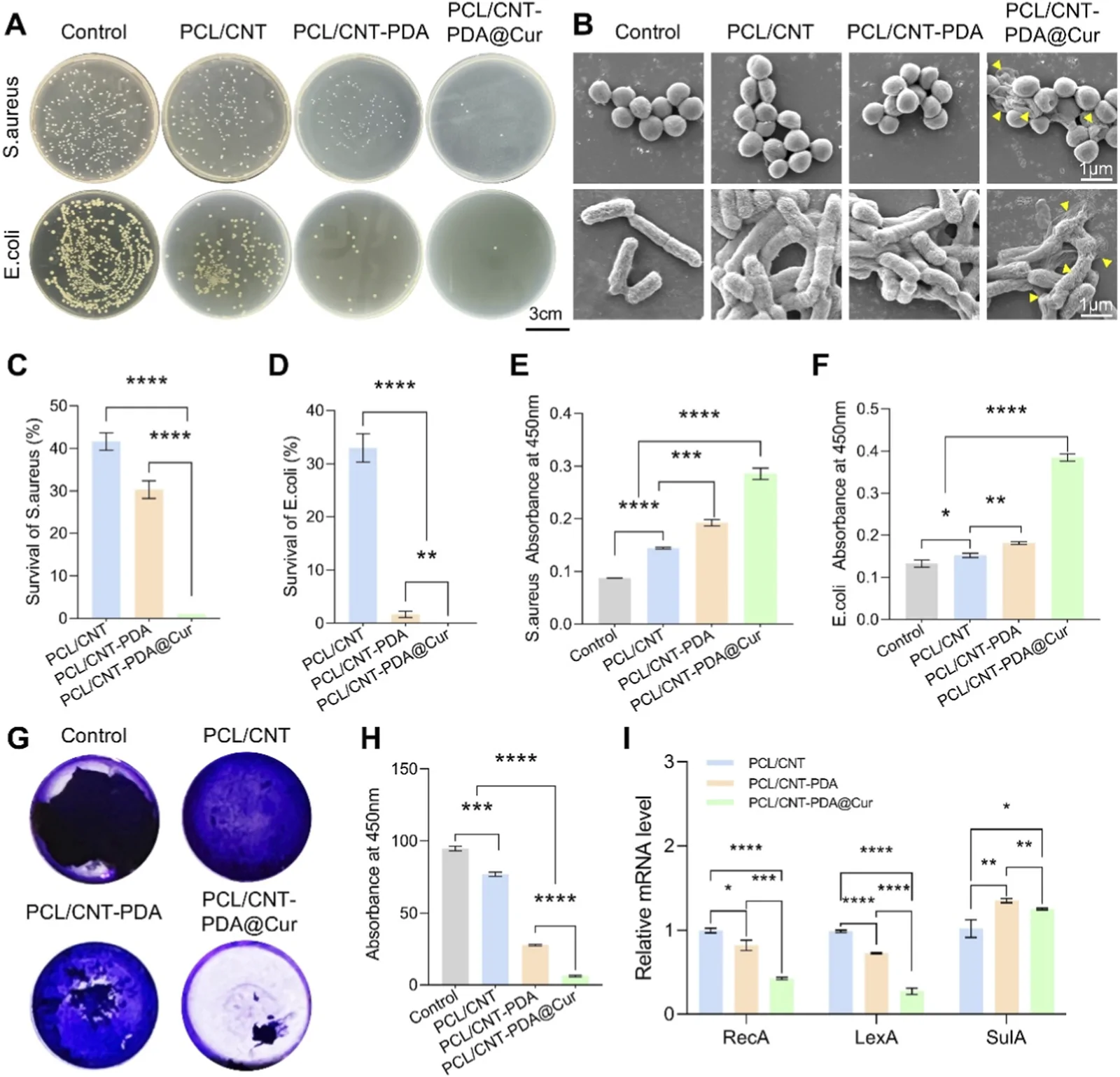
**(A)** Colony formation of *Staphylococcus aureus* and *Escherichia coli* after treatment with different fibrous scaffolds; **(B)** Scanning electron microscopy (SEM) images of *Staphylococcus aureus* and *Escherichia coli* after treatment with different fibrous scaffolds (yellow arrows indicate damaged bacteria); **(C, D)** Survival rates of *Staphylococcus aureus* **(C)** and *Escherichia coli* **(D)** after treatment with different fibrous scaffolds (n = 3); **(E, F)** Membrane permeability of *Staphylococcus aureus* **(E)** and *Escherichia coli* **(F)** after treatment with different fibrous scaffolds (n = 3); **(G)** Crystal violet staining of bacterial biofilms; **(H)** Quantitative analysis of biofilm formation based on crystal violet staining (n = 3); **(I)** Relative expression levels of key genes involved in the SOS response, including *RecA*, *LexA*, and *SulA* (n = 3). All data are presented as mean ± SD, and statistical significance was determined by one-way ANOVA followed by Tukey’s *post hoc* test; *p < 0.05, **p < 0.01, ***p < 0.001.

The formation of bacterial biofilms is a key factor contributing to chronic infections and drug resistance (Liu et al., 2024). Bacterial membrane permeability assay results (Figures 4E,F) showed that the absorbance of ONPG hydrolysis products in the PCL/CNT-PDA@Cur group was significantly higher than that in the PCL/CNT group, indicating that the scaffolds significantly increased bacterial cell membrane permeability. This finding is consistent with the membrane damage observed by SEM, confirming that curcumin likely exerts its antibacterial effects through disruption of bacterial membrane integrity. Crystal violet staining (Figure 4G) and quantitative analysis (Figure 4H) of *S. aureus* biofilms further demonstrated that the PCL/CNT-PDA@Cur scaffolds significantly inhibited bacterial biofilm formation, with crystal violet absorbance values significantly lower than those of the PCL/CNT group.

Bacterial qPCR results (Figure 4I) revealed the interference of the scaffolds with the bacterial SOS response system. The SOS response is a self-protection mechanism activated by bacteria in response to DNA damage and other stressors, regulated by key genes including *RecA* and *LexA*. The data showed that the PCL/CNT-PDA@Cur scaffolds significantly downregulated the mRNA expression levels of *RecA* and *LexA*.

Notably, there may be a synergistic relationship between the downregulation of SOS genes and the high bactericidal rate of the scaffold: on one hand, the scaffold directly causes leakage of bacterial contents and cell death by disrupting bacterial membrane integrity (Figure 4B); on the other hand, the suppression of the SOS repair pathway deprives bacteria of a key defense mechanism against membrane damage and oxidative stress, and the synergy between these two effects significantly enhances the bactericidal outcome. However, the precise molecular mechanism underlying SOS gene downregulation still requires further elucidation—it remains unclear whether curcumin directly inhibits RecA protein ATPase activity or DNA-binding capacity, or whether oxidative stress induced by the scaffold causes bacterial DNA damage that indirectly affects the SOS pathway through feedback regulation. Bellio et al. have reported that curcumin inhibits levofloxacin-induced SOS response in *E. coli*, suggesting the possibility of direct interference with RecA function. Notably, in the present study, the SulA gene showed an upregulation trend. As an effector protein of the SOS response, SulA upregulation may represent a feedback stress response following suppression of the RecA-LexA regulatory axis, or a compensatory response to DNA damage induced by the scaffold. This phenomenon is consistent with the complex regulatory pattern of curcumin on the SOS response reported in the literature (Bellio et al., 2014). However, the contribution of oxidative stress in the present system should not be overlooked. Future studies employing RecA ATPase activity assays, bacterial intracellular ROS quantification, and comet assays for DNA damage evaluation are warranted to delineate the precise molecular pathway.

### 3.6 *In vivo* wound healing assessment

The *in vivo* wound healing efficacy of the PCL/CNT-PDA@Cur scaffolds was further validated in a rat full-thickness skin defect infection model. Wound healing was monitored and recorded on post-operative days 0, 3, 7, and 14.

Representative wound photographs (Figure 5A) showed that on day 7, the wound area in the PCL/CNT-PDA@Cur group was markedly smaller than that in the other groups. By day 14, the wounds in the PCL/CNT-PDA@Cur group were almost completely closed, with a closure rate significantly higher than that of the other groups, whereas the control group still exhibited large unhealed wounds. Quantitative analysis of wound closure rates (Figures 5B,C) revealed that on days 3, 7, and 14, the wound closure rates in the PCL/CNT-PDA@Cur group were significantly higher than those in the PCL/CNT and control groups (*P* < 0.01), indicating that this scaffold markedly accelerated the wound healing process. Figure 5A bacterial plate culture of wound tissues and quantitative bacterial survival rate analysis (Figure 5E) confirmed that the PCL/CNT-PDA@Cur scaffolds also exhibited potent antibacterial efficacy *in vivo*, effectively controlling wound infection.

**FIGURE 5:**
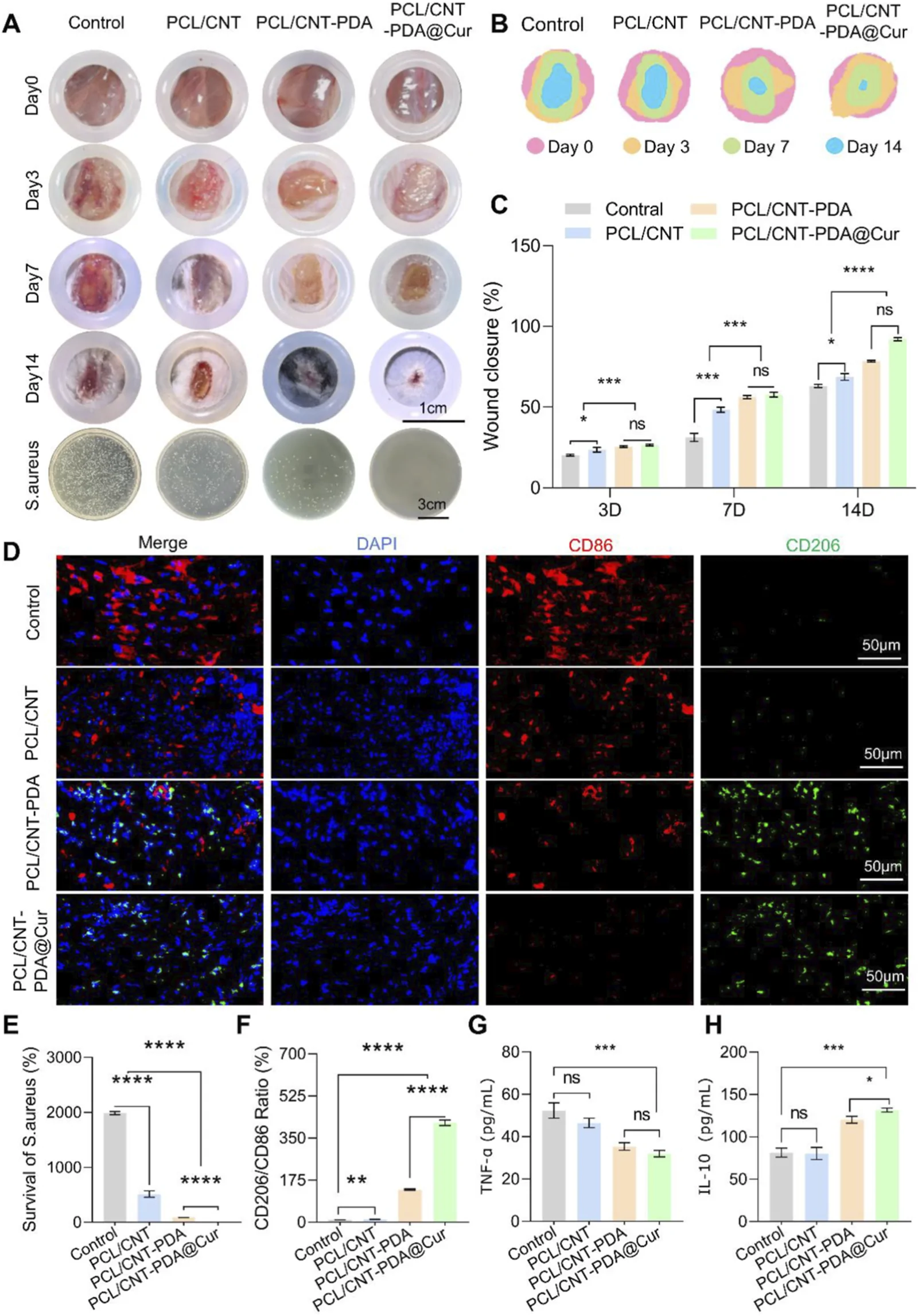
**(A)** Representative photographs of wounds in different treatment groups on post-operative days 0, 3, 7, and 14, along with plate culture analysis of *Staphylococcus aureus*-infected wounds (n = 10); **(B)** Quantitative analysis of wound area in different treatment groups (n = 10); **(C)** Wound closure rates of different groups on days 0, 3, 7, and 14 (n = 10); **(D)** Immunofluorescence staining of CD86, CD206, and DAPI in regenerated wound tissues on day 7 (n = 5); **(E)** Bacterial survival rate (n = 3); **(F)** Quantitative analysis of the CD86/CD206 immunofluorescence intensity ratio (n = 5); **(G, H)** ELISA analysis of TNF-α **(G)** and IL-10 **(H)** in regenerated wound tissues on day 7 (n = 5). All data are presented as mean ± SD, and statistical significance was determined by one-way ANOVA followed by Tukey’s *post hoc* test; *p < 0.05, **p < 0.01, ***p < 0.001.

To assess the inflammatory state of the wound microenvironment, the phenotypes of macrophages and the expression of inflammatory cytokines in wound tissues on day 7 were examined by immunofluorescence staining. The results (Figures 5D,F) demonstrated that in the PCL/CNT-PDA@Cur group, the expression of the M2 macrophage marker CD206 was significantly higher than that in the other groups, while the expression of the M1 marker CD86 was significantly reduced, resulting in a markedly increased CD206/CD86 ratio (P < 0.01). ELISA analysis (Figures 5G,H) further revealed that in the PCL/CNT-PDA@Cur group, the level of the pro-inflammatory cytokine TNF-α was significantly decreased, whereas the level of the anti-inflammatory cytokine IL-10 was significantly increased (P < 0.01), indicating the establishment of an immune microenvironment favorable for tissue repair. These results are consistent with the *in vitro* macrophage polarization findings, confirming that the scaffold effectively regulates the wound immune microenvironment *in vivo*, promotes inflammation resolution, and facilitates tissue repair.

## 4 Histological evaluation of wound healing

To further evaluate the wound healing efficacy, histological analysis of wound tissues was performed on post-operative days 7 and 14. H&E staining results (Figure 6A) showed that on day 14, the wound length in the PCL/CNT-PDA@Cur group was significantly smaller than that in the other groups (Figure 6B), while the granulation tissue thickness was significantly greater (Figure 6C). Notably, obvious hair follicle-like structures were observed in the regenerated tissues of the PCL/CNT-PDA@Cur group. Masson’s trichrome staining (Figure 6D) revealed that the PCL/CNT-PDA@Cur group exhibited the most abundant collagen deposition with more orderly arranged collagen fibers. Quantitative analysis of collagen content (Figure 6E) indicated that the percentage of collagen-positive area in this group was significantly higher than that in the other groups (*P* < 0.01), suggesting that the scaffold promoted skin regeneration. However, Masson staining only reflects total collagen content, and future studies combining Col I/III immunohistochemical staining are needed to further evaluate collagen maturity.

**FIGURE 6:**
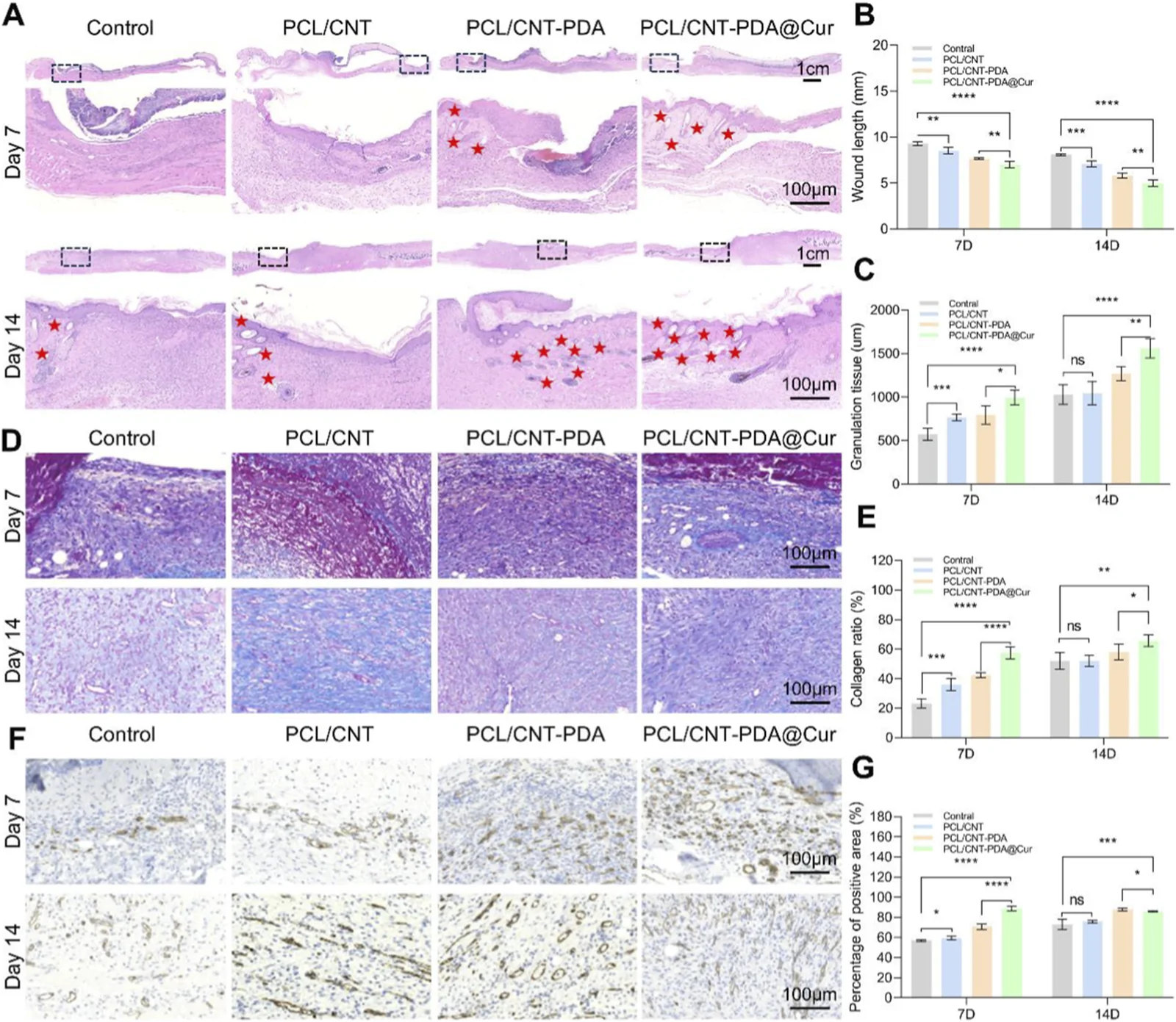
**(A)** Representative H&E staining of regenerated tissues on post-operative days 7 and 14 (asterisks indicate hair follicles); **(B)** Quantitative analysis of wound length (n = 10); **(C)** Quantitative analysis of granulation tissue thickness (n = 10); **(D)** Representative Masson’s trichrome staining of regenerated tissues on post-operative days 7 and 14; **(E)** Collagen content in regenerated wound tissues (n = 5); **(F)** Representative CD31 immunofluorescence staining of regenerated tissues on post-operative days 7 and 14; **(G)** Quantitative analysis of CD31 expression in regenerated wound tissues (n = 5). All data are presented as mean ± SD, and statistical significance was determined by one-way ANOVA followed by Tukey’s *post hoc* test; *p < 0.05, **p < 0.01, ***p < 0.001.

CD31 immunofluorescence staining results (Figure 6F) demonstrated that on day 7, the percentage of CD31-positive area in the PCL/CNT-PDA@Cur group was significantly higher than that in the other groups (*P* < 0.01), indicating that the introduction of curcumin effectively promoted early angiogenesis. By day 14, the CD31-positive area in the control, PCL/CNT, and PCL/CNT-PDA groups continued to increase, whereas that in the PCL/CNT-PDA@Cur group slightly decreased. This observation does not indicate weakened angiogenic capacity of this group; rather, it reflects the faster wound healing progression in this group, which had already completed re-epithelialization and tissue remodeling by day 14, entering the late repair phase characterized by physiological regression of the capillary network. Quantitative analysis of the CD31-positive area percentage (Figure 6G) confirmed this difference (*P* < 0.01). These results are consistent with the *in vitro* HUVEC tube formation findings, further confirming the pro-angiogenic ability of the scaffold and its capacity to accelerate the transition from the proliferative phase to the remodeling phase, thereby achieving faster overall healing.

Integrating the *in vivo* results from Figures 5, 6, a complete logical chain for PCL/CNT-PDA@Cur scaffold-promoted wound healing can be outlined. First, at the level of inflammatory regulation (Figures 5D–H), the scaffold promotes macrophage polarization toward the M2 anti-inflammatory phenotype, significantly reducing pro-inflammatory TNF-α levels and increasing anti-inflammatory IL-10 levels, thereby shifting the wound microenvironment from a pro-inflammatory state to an immune microenvironment conducive to tissue repair. This transition is a prerequisite for subsequent repair processes. Second, at the level of angiogenesis (Figures 6F,G), the scaffold significantly increases CD31-positive area on day 7 through the direct pro-angiogenic effects of curcumin and the indirect effects of M2 macrophage-secreted factors such as VEGF, providing sufficient nutrition and oxygen supply for granulation tissue formation. Notably, the slight decrease in CD31-positive area in the PCL/CNT-PDA@Cur group on day 14 reflects the earlier entry of this group into the tissue remodeling phase—newly formed capillaries undergo physiological regression after completing their repair mission. Finally, at the level of tissue remodeling (Figures 6A–E), the abundant vascular network supports robust granulation tissue formation and orderly collagen deposition, and the hair follicle-like structures observed exclusively in the PCL/CNT-PDA@Cur group on H&E staining further confirm the high-quality skin regeneration achieved by this scaffold. In summary, the *in vivo* data support a complete therapeutic cascade: immune microenvironment remodeling (Figure 5) → enhanced angiogenesis (Figure 6) → tissue regeneration and remodeling, with these three elements interlinked to collectively drive rapid and high-quality wound healing.

It should be noted that although the rat full-thickness skin defect model used in this study is a commonly employed *in vivo* evaluation system in tissue engineering, wound closure in rats relies heavily on panniculus carnosus contraction, which differs from the healing mechanism in human skin that primarily involves granulation tissue formation and re-epithelialization. Therefore, the *in vivo* results of this study should be interpreted with caution when extrapolating to clinical translation. Although H&E staining showed significantly shortened wound length in the PCL/CNT-PDA@Cur group (Figure 6B), indirectly reflecting accelerated re-epithelialization, this study did not perform independent quantitative analysis of re-epithelialization rate. Future studies should further validate the healing efficacy of this scaffold in large animal models such as pigs, whose skin structure more closely resembles that of humans, and conduct systematic quantitative assessments of re-epithelialization rate and collagen maturity.

In summary, the multifunctional electroactive PCL/CNT-PDA@Cur scaffold constructed in this study can effectively modulate the wound microenvironment and accelerate skin wound healing through the synergistic integration of multiple functions, including antioxidant, anti-inflammatory, antibacterial, and pro-angiogenic activities.

## 5 Conclusion

In this study, a multifunctional electroactive nanofibrous scaffold loaded with curcumin (PCL/CNT-PDA@Cur) was successfully constructed via electrospinning combined with polydopamine-mediated surface functionalization, and its application potential in wound repair was systematically evaluated.

The scaffold exhibited oriented fibrous architecture, favorable hydrophilicity, stable electrical conductivity, and sustained curcumin release properties. *In vitro* studies demonstrated that the PCL/CNT-PDA@Cur scaffold significantly promoted the proliferation of L929 fibroblasts and the expression of collagen types I and III, enhanced the tube-forming capacity of HUVECs and the expression of angiogenesis-related genes, while effectively scavenging intracellular reactive oxygen species and protecting cells against oxidative stress-induced apoptosis. The scaffold also modulated RAW264.7 macrophage polarization toward the anti-inflammatory M2 phenotype and exhibited potent antibacterial activity against *Staphylococcus aureus* and *Escherichia coli* by disrupting bacterial membrane integrity. Furthermore, a novel antibacterial mechanism was uncovered, involving the downregulation of *RecA* and *LexA* genes to inhibit the bacterial SOS response. In a rat full-thickness skin defect infection model, the PCL/CNT-PDA@Cur scaffold effectively controlled infection, significantly accelerated wound closure, and promoted granulation tissue formation, collagen deposition, and neovascularization. It also improved the wound immune microenvironment and facilitated the regeneration of skin appendages.

Collectively, the PCL/CNT-PDA@Cur scaffold achieves effective remodeling of the wound microenvironment through the integration of multiple functions—including physical structural guidance, electroactive conduction, antioxidant activity, immunomodulation, antibacterial effects, and pro-angiogenic properties—thereby offering a promising strategy for the treatment of complex wounds. This work provides a versatile and integrated dressing platform with great potential for managing chronic and refractory infected wounds.

## Data Availability

The original contributions presented in the study are included in the article/Supplementary Material, further inquiries can be directed to the corresponding authors.
