# Supplementary material for: Polydopamine-mediated curcumin-functionalized electroactive nanofibrous scaffold accelerates infected wound healing via multifactorial microenvironment remodeling
Source: Front Bioeng Biotechnol. 2026 Oct 14;14:1959216. doi: 10.3389/fbioe.2026.1959216 (PMC13713858; doi:10.3389/fbioe.2026.1959216)
Supplement: Supplementary file 1 [file Supplementaryfile1.docx]

**Supporting Information**

**Figure S1.** Proliferation of HUVEC cell on PCL/CNT, PCL/CNT-PDA, and PCL/CNT-PDA@Cur fiber scaffolds assessed by CCK-8 assay at 1, 3, and 5 days (n=3).

**Figure S2.** Proliferation of L929 cell on PCL/CNT, PCL/CNT-PDA, and PCL/CNT-PDA@Cur fiber scaffolds assessed by CCK-8 assay at 1, 3, and 5 days (n=3).
